# A Memoir on Those Diseases Which Proved so Fatal to Our Troops during the Burman War; with a Comparative Sketch of Their Analogous Cases in Hindostan, during a Service of Some Years

**Published:** 1829-01

**Authors:** James Walsh

**Affiliations:** Assistant Surgeon 89th Regiment.


### DISEASES IN HINDOSTAN.

*A Memoir on those Diseases which proved so fatal to our Troops during the Burman War; with a comparative Sketch of their analogous Cases in Hindostan, during a Service of some years.* By James Walsh, Assistant Surgeon 89th Regiment.

Medico-tropical investigation has been so ably and luminously exercised by Drs. Chisholm, Johnson, and several others, that it should not have been ventured upon by me, if the circumstances in which I was placed in Burmah did not afford some peculiar facilities for comparative effect.

Thus guided, therefore, in my examination of ordinary tropical disease, I had but to follow those master minds, who have so successfully traversed that field of inquiry as to leave little for further research. This summary of Burman pathology, exhibiting no small diversity of character, is the result of attentive observation, and, I may be permitted to add, of long, painful, and protracted suffering; having laboured under almost every form of disease, and perhaps every violence of symptom, as endeavoured to be portrayed.

In endeavouring to account for the frightful mortality which occurred during the Burman war, a slight medico-topographical sketch of the country, as well as of the peculiar circumstances to which the force, both naval and military, was exposed, may be considered necessary for the better conception of its nature.

A comparison, also, of the more violent diseases under which our troops suffered, with those of Hither India, whose more prominent symptoms hold some correspondence, may afford, perhaps, further elucidation.

Of the Indian peninsula, and its sources of morbific action, so much has been written, and so ably, that its topography need scarcely be adverted to, unless for comparative effect with the recent seat of war. The diseases of India, citra, as well as extra Gangem, evince as marked an analogy as their respective localities and parallels of latitude would admit of. Whenever deviations took place from the ordinary course of morbid action, they might, perhaps, with more justice be ascribed to those modifications necessarily arising in a rapid transition from the wholesome diet, comfortable accommodation, vigilant superintendence, and minute attention to their wants, enjoyed by the soldiery when in cantonments in Hindostan; whilst in Burmah there existed a miserable and melancholy reverse.

The river Burrampeetu, from its source in the southern extremity of the Himalayan mountains to its union with the Ganges, or termination in the Megna, would appear to form a more correct line of boundary between India, properly so called, and that portion of Indo-China constituting the Burman empire, as it existed before our territorial acquisitions in that quarter. This line of division with the great Indian Delta, running W. and S.W. somewhat parallel to that mountain chain, extending in the same direction from the Imaus, in unbroken yet diversified continuity, presents nearly the same morbific causes, the same field for investigation, as the Irriwaddy, or great river of Ava. This river rises at no great distance from the former, and pursues a course nearly similar, but at the eastern side of that chain throughout its whole extent, under its subdivisional names of the Assam, Cachar, Tipperah, Aracan, and Kiayn mountains. The Irriwaddy, soon after its arrival in Pegu, sends out many branches, communicating and ramifying in amazing diversity throughout that province, forming a delta and interprovincial navigation much superior in extent to that of the Megna.

These two great rivers, in their relative course, receive numerous streams from the above-mentioned series of mountains, yet their waters exhibit occasionally a marked difference. That of the Burrampootu, at many points of its course, after standing a certain time, becomes clear, soft, and well tasted, and neither argil, lime, nor mica are found in the deposition; whilst the water of the Irriwaddy, particularly after the commencement of the rains, will have all three, and cannot be thoroughly cleared by subsidence or filtration, generally having a lightish, milky colour. The banks of this river are formed of alluvian strata in very considerable diversity, calcareous earth, pipe clay, and schistose alumina, with a variety of soils deposited in layers according to their respective gravities. The banks of the Burrampootu appear of a more uniform deposition of common earth, and without any trace of petrifactive property in its waters; whilst the banks and bed of the Burman river will exhibit for miles little other stony concretion than those formed from the petrifaction of the various woods of that region, and occasionally met with in masses of some magnitude, the petrifaction retaining the true fibrous appearance of the original wood, although differing so much in specific gravity and density.

These great deltas, forming diversified alluvion to a vast extent, and covered with wood and jingle in nearly equal density, exhibit considerable uniformity of morbific action; yet a marked deviation has been occasionally manifested in the difference of type and degree of mortality, particularly in dysentery and remittent fever. This modification of disease could not have arisen from the geographical difference merely of a few degrees of latitude or longitude, whilst their respective peculiarities of climate, products, &c. bore a striking analogy. It must, therefore, be sought for in those circumstances alluded to before, to which the expeditionary force was so unfortunately and unseasonably subjected.

The expedition was undertaken at the beginning of the S.W. monsoon, or rainy season: of course, little further could be achieved than the capture and occupation of Rangoon, and afterwards guarding against a coup de main from the numerous parties of the enemy, entrenched at various points and distances, but gradually concentrating about our position.

Divisions and detachments were, now and then,—once, twice, or perhaps three times a week,—ordered into the jungle, either for reconnoisance or attack, during the most tremendous monsoon recollected there, without roads, bridges, or landmarks of any description, to assist or guide them in the almost general inundation pervading the low country.

For the first four months, the troops had not merely to grope their way, or wade above the hips or knees, for ten or twelve miles, floundering or rolling, perhaps, in the mud and water half the way, but were occasionally obliged to swim; and this frequently without meeting an enemy; but, even when found and driven from his entrenchment, it was discovered to be reoccupied and restockaded in two or three days. The same routine of wading, swimming, and attack, was therefore to be repeated, with little other possible results than the rapid diminution of our force by disease or death; a result, indeed, upon which the Burmans were known to contemplate with exulting certainty.

The occupation of the great pagoda (Shoe-dragon), about three miles N. of Rangoon, called for the support of the greater part of the force, in two or more lines, including a large park of artillery nearly in their centre. The troops, principally European, thus stationed, were under the necessity of proceeding to Rangoon, in greater or less numbers, once or twice a day, for their rations, exposed to the alternate deluges of rain and a vertical sun; consequently, subjected in a few hours to sudden and frequent changes of temperature, perhaps from 70 to 76° Fahrenheit to 100 or 120°, when the sun had for some time burst upon them clear and unclouded.

It can require but little divination to foresee the consequences of this state of things. The men sickened in great numbers, notwithstanding all the precautions, regimental or medical, that could be adopted; and at times but few of the officers could be found fully capable of their ordinary duty. At length a medical representation was forwarded, and the services of the public porters and vehicles were directed to be extended, which had been previously confined to the accommodation of one or two corps only.

The seeds of morbid action, thus unsparingly laid, quickly burst forth with great violence: sickness, and a mortality amounting to comparative annihilation, soon took place; the deaths in the 89th sometimes averaging 100 per week, and were exceeded in other corps. The sum total of deaths for the first three months, exclusive of casualties in action, trifling in point of number, must have been upwards of 3000 Europeans, or more than half the force originally despatched.

During this melancholy visitation, the rations of the soldier were poorly calculated to withstand disease, or strengthen a convalescence rather generally precarious and doubtful. The biscuit was frequently rotten, mouldy, and so full of large white maggots and other insects as to be now and then nearly capable of locomotion. The beef and pork often so rancid as not to be freed by any degree of washing from the offensive stench and numerous animalculæ, in a state of incessant reproduction and decay. The rice also at times damaged and unsound; and the spirit served out (rum or arrack) rather in a state of too great dilution to excite sufficient energy in the soldier, sinking under extreme fatigue, or a low and protracted convalescence.*

The human frame (European) in a tropical climate, particularly in India, is always in a state of such high susceptibility, as to be powerfully acted upon by those morbific causes which are in a state of perpetual action, although modified occasionally into no small diversity of character. The endemic becomes epidemic, often with an increased exasperation of type; or may undergo a total change, and exhibit disease of a varied, or perhaps wholly opposite, description. How else is that rapid transition, occurring in Burmah, to be accounted for, wherein bilious remittent subsided, and was immediately succeeded by rheumatic or intermittent fever, dysentery or cholera, too often to so violent a degree as could not have been expected after the gradual amelioration of those circumstances, so inadequately sketched, yet productive of such terrific results. It is true vegeto-animal miasm may have acquired a more specific agency, and a higher degree of concentration, than it possessed on our arrival, and, of course, act with a virulence proportioned to this increased power, to which that extreme debility and exhaustion must have not a little contributed, which supervened on such a combination of untoward circumstances, and to which the whole force was so perseveringly exposed.

The principal diseases of this period, while military operations were confined to the great delta of Pegu, were remittent, intermittent, and rheumatic fevers, with cholera and dysentery. The two former sometimes alternating, almost always violent, and often fatal: at least such was the general result in those of later occurrence; the rheumatic never so, but rapidly merging into an extremely troublesome chronic state, and rendered particularly obstinate from the existing necessity of frequent exposure to wet, when perhaps under mercurial influence; a necessity becoming every day more imperious, and calling so particularly for individual exertion,

* To the credit of the Madras local government, it should be said, that, as soon as this want of equipment, and its sad effects, were known there, a large supply of every requisite for comfort and the restoration or preservation of health was immediately furnished, and most liberally distributed among the hospitals of its division.

in consequence of the increasing prevalence of illness throughout every part of the force.

The bilious remittent did not always evince an equal degree of violence. In some early cases it soon intermitted, or wholly subsided, without manifesting a high range of excitement, febrile or cerebral, and yielding in a few days to purgatives, pediluvium, saline diaphoretics, and cold applications to the head, with occasional cold affusion, topical and general. But those of later occurrence, or relapsed cases, were attended with different symptoms and results; generally high febrile excitement and sensorial disturbance. In the primary and severe cases of this nature, the head was always affected with excruciating pain, at first in the frontal and temporal regions, and gradually propagated to the occipital and parietal. This was accompanied with a perceptible pulsatory action of the cranial contents, apparently the cause of a feeling of excessive soreness to the touch in its integuments and external surface. The eyes, to a very considerable degree, partook of this excessive disturbance in their immediate neighbourhood and connexions, soon becoming inflamed, painful, protruding, and intolerant of light.

This determination to the head, generally setting in with such rapidity as not to be marked with any obvious premonitory symptoms, can only be attributed to some powerful agent on the animal economy, to the operation of which the variety of predisposing causes glanced at before must materially, although in a varied degree, have contributed. This agent must therefore be looked for in the constitution of the atmosphere, impregnated, to various degrees of concentration and malignity, with vegeto-animal miasm or effluvia, from vegetable and animal matter in a state of putrefaction and decay, to the more perfect evolution or formation of which heat and moisture are indispensable.

The immediate effect of this invisible poison is, I believe, universally admitted to be sedative: hence the cerebral disturbance, and consequent derangement of function, throughout the nervous system. The balance of the circulation is soon broken, of course, and more or less of congestion, local inflammation, or other similar topical affection, is likely to take place, as a natural result of the miasmal influence, and the violent reaction too often immediately supervening. The healthy balance of the system thus broken, and succeeded by a numerous train of symptoms indicative of extreme irregularity and excitement in some organs, whilst others are struck torpid and rendered incapable of their appropriate function: the speediest means of relief must therefore be had recourse to, or the animal frame must soon sink under this complicated and overwhelming mischief. The derangement of the sensorial functions is accompanied, or quickly followed, by corresponding efforts on some of the abdominal organs; at least, those connected with the chylopoietic viscera. Derangement and congestion in these would appear to have arisen synchronously with the cerebral, or at least to advance with them *pari passu*, but operating, perhaps, in a different manner. The heart and arterial circulation, at all times easily roused, even by a very trifling or momentary cause, cannot remain long unaffected in this state of general disturbance: hence the violent impetus to the head, and "remora in the portal circle;" the one in a state of inordinate excitement, whilst the chief organ of the latter is in a state of torpor.

The excruciating pain of the head and affection of the eyes, as well as the excessive heat and pain about the præcordia, may thus be accounted for.

The first step, then, in the *methodus medendi* can scarcely be mistaken in this stage of the complaint; and, if the lancet is not most freely, perhaps repeatedly used, the destruction of those organs must be the inevitable result. Bleeding, therefore, *ad deliquium*, as freely and rapidly as possible, in the first few hours of attack or state of violent reaction, must be practised, and, if not attended with an evident diminution, as well as general relief of the more urgent symptoms, be repeated as soon as the absence of syncope and return of excitement would admit of or call for it.

From this view, it must be obvious that the abstraction of blood in the first instance, to as great an extent as possible, can afford us the only chance, at least, of immediately relieving the head and præcordia; and, if this is not procured, all else must be vain. There is no article in the materia medica capable of acting with the requisite promptitude. Some may serve as powerful auxiliaries, but their modus operandi must be too circuitous to arrest the rapid disorganization, perhaps already begun, or save from destruction organs of vitality so overpowered. This depletion of the system, and reduction of the phlogistic diathesis, will pave the way, and may give time, for the successful operation of other medicines. Cerebral excitement is not only likely to be relieved, and hepatic congestion diminished, but the function of this organ partially renewed, although perhaps rather irregularly, or not with a sanatory effect.

A frequent discharge of bile, more or less vitiated, during the gastric irritability so generally attendant on cases of this nature, is an invariable accompaniment; as is also the altered state of the secretions and skin, arising from derangement of this viscus, with perhaps some other morbid action not yet ascertained, sometimes giving to the disease the more marked features of the great western endemic. Hence the necessity for the exhibition of purgatives, and the full and speedy excitement of mercurial action.

The utility of purgatives, so decisively and generally established, is in no case more manifest than in tropical fever. The impetus of blood and congestion in the portal circle must thereby undergo a greater or less diminution, as a necessary consequence of the increased determination to the intestinal canal.

From the peculiar action of mercury on the glandular system, its conjoined and separate use should be carefully followed up, not merely as a beneficial adjuvant in the ordinary course of purgatives, but so as to produce its proper and full impregnation of the system as soon as possible. Whether it might be inferred that the febrile action would subside as the mercurial advanced, from their supposed incompatibility, or whatever its modus operandi may be, still the benefits arising from its exhibition are long established and unquestionable.

For the production of these beneficial results, certain circumstances and a certain state of the system must always be held in view. Too high a degree of phlogistic excitement, or its opposite, of great exhaustion, are extremely unfavorable to the action of this medicine on the human frame. Without this action (excited as rapidly as possible), as powerfully auxiliary to bleeding and purgatives, the disorganization of some viscus, and destruction of the patient, must be an almost inevitable and speedy consequence, unless relieved considerably by the prior evacuation.

The first of these circumstances, the phlogistic diathesis, may be supposed enough diminished by the free primary depletion. The second, or state of exhaustion, not likely to occur, unless in protracted cases, must be combated by appropriate stimuli, so as to rouse the absorbents to their requisite action. The consideration, therefore, of these two vital points call for serious discrimination and sound practical acquirement.

In the progress of the disease, some minor articles may also be brought to our assistance, such as cold affusion, if admissible, topically or generally applied, at least cold applications to the head freed from its hair; saline diaphoretics, sinapisms, pediluvium; obstinate remains of local affection to be treated by friction, fomentation, cupping, blisters, &c. On the disappearance of the inflammatory symptoms, and subsidence or remission of febrile excitement, the debility is to be counteracted by a gradual and well-regulated course of tonics and regimen.

When motion is admissible, a change of scene and climate should take place as soon as possible, and those modes of gestation practised which are most agreeable; such as sailing, use of a carriage, equitation, &c. Indeed, on the approach of the complaint, thorough ventilation should not only be carefully attended to, but the removal of the patient to a purer, or even another atmosphere, should, if practicable, take place; as, although remittent fever may have fully formed, he is still exposed to an increased degree of exasperation, if kept within the influence of the exciting cause. This removal must also afford a better chance of favorable action for medicine, and ultimately of convalescence, contrary to the opinion "that the remote causes of fever cease to act as soon as the disease has fully developed itself."*

Emetics, which have been heretofore much used in all febrile cases, are now rather generally rejected, at least in tropical ones, of course are omitted in the series of medicines already enumerated as likely to be injurious in most stages of bilious remittents, whilst the impetus of blood to the head continues undiminished, or when the gastric irritability is excessive. This latter must be increased by the violent straining so likely to occur; as must also the determination to the head, from the same cause; whilst, on the other hand, antimonials given so as to cause a slight nausea, or combined with calomel to produce a specific or febrifuge effect, sometimes assisted advantageously in lowering the high tone of the system. But still antimonials, particularly as emetic, would appear to be rather universally contraindicated in tropical disease, as giving rise frequently to even a considerable degree of spasm, perhaps cholera, after the convulsive straining, so apt to run into excess, and, of course, add to the debility already existing. Saline cathartics, at least in a full dose, were found rather inadmissible, from the apprehension of a similar effect.

* Those conclusions of Dr. James Johnson I found to be correctly established in a great number of cases, particularly my own, wherein it was attended with very marked results. A more perfect remission almost immediately taking place on my removal, when labouring under jungle remittent, as also a diminution of the more prominent symptoms, and speedy change of type to the intermittent, which, after considerable irregularity and severity, yielded to cinchona, with purgatives and mercurials: the latter obliged to be had recourse to from some remaining unsubdued hepatic uneasiness.

This did not particularly strike me till pointed out by the late surgeon* of the corps in which I have served some years.

The primary or secondary intermittents of the Pegu delta evinced, at their commencement, nearly the same general characters as are observed in those of Bengal, yet modified in their subsequent progress by miasmal concentration; to which also unsound rations, great exposure, and fatigue, must have powerfully contributed.

After a short time, the periods of intermission might be observed extended or curtailed, and throughout attended with irregularity to such a degree as to confound, more or less, their classification. The transition, also, from one stage to another, or their unequal duration and mixed symptoms, led, in the beginning, to much embarrassment on the part of the practitioner, as well as increased and often protracted suffering to the patient. The accessions or duration of the paroxysms were seldom certain, more frequently changing so abruptly or irregularly as to prevent the recognition of the decided tertian, quartan, or quotidian. Sometimes the disease would, for the space of an interval or two, assume the form of the virulent remittent, with great cerebral disturbance and epigastric or hypochondric uneasiness. Even the more steady intermittents were often marked with a degree of violence not unfrequently defeating every effort of the medical officer.

In the early stages of the more violent and irregular, a course of treatment was followed as similar to that pointed out for the remittent, on its formation, as the train of symptoms, general and local, would admit. Where the system was not much broken down, and that cerebral or other local determination, with pyrexia, still continued, bleeding, both topical and general, should have been practised to as great an extent as the state of the pulse, degree of excitement, and general strength would allow. Mercurials and purgatives were followed up, nearly as pointed out under the head of remittents. When the local determination and disturbance showed any considerable reduction, recourse was had to cold

* Dr. DAUN, a medical officer long resident in India, of profound research and great practical acquirement, whose talents for observation was not more marked and unceasing than its very successful application, wholly unfettered as it was by any preconceived theory or unfounded bias. To him I feel myself strongly called on for my individual acknowledgments, owing, as I do, to his experience and inculcation, not merely my own existence, after repeated attacks of disease, but also the satisfaction of witnessing the benefits resulting from the application of principles so evidently founded on just medical inference and sound logical deduction.

affusions, anodynes, tonics, and corroborants, at the proper periods for their exhibition.

There was occasionally a feeling of intense anxiety and alarm, bordering on terror, so strongly marking the Pegu intermittent, and apparently unconnected, from its peculiarity of sensation, with any existing topical or general excitement, as to call for the exhibition of opiates to a very liberal extent, if not strongly contraindicated.

[To be continued.]

---

